# Fasting breath H_2_ and gut microbiota metabolic potential are associated with the response to a fermented milk product in irritable bowel syndrome

**DOI:** 10.1371/journal.pone.0214273

**Published:** 2019-04-04

**Authors:** Boris Le Nevé, Muriel Derrien, Julien Tap, Rémi Brazeilles, Stéphanie Cools Portier, Denis Guyonnet, Lena Ohman, Stine Störsrud, Hans Törnblom, Magnus Simrén

**Affiliations:** 1 Danone Nutricia Research, Palaiseau, France; 2 Diana Nova, Clichy La Garenne, France; 3 Department of Immunology and Microbiology, Institute of Biomedicine, Sahlgrenska Academy, University of Gothenburg, Gothenburg, Sweden; 4 Department of Internal Medicine and Clinical Nutrition, Institute of Medicine, Sahlgrenska Academy, University of Gothenburg, Gothenburg, Sweden; 5 Center for Functional Gastrointestinal and Motility Disorders, University of North Carolina, Chapel Hill, North Carolina, United States of America; Wageningen University, NETHERLANDS

## Abstract

**Objectives:**

Aim of this study was to assess the effect of a fermented milk product containing *Bifidobacterium lactis* CNCM I-2494 (FMP) on gastrointestinal (GI) symptoms and exhaled H_2_ and CH_4_ during a nutrient and lactulose challenge in patients with irritable bowel syndrome (IBS).

**Methods:**

We included 125 patients with IBS (Rome III). Fasted subjects were served a 400ml liquid test meal containing 25g lactulose. The intensity of eight GI symptoms and the amount of exhaled H_2_ and CH_4_ were assessed before and during 4h after meal intake. The challenge was repeated after 14 days consumption of FMP or a control product in a double-blind, randomized, parallel design. The metabolic potential of fecal microbiota was profiled using 16S MiSeq analysis of samples obtained before and after the intervention.

**Results:**

106 patients with IBS were randomized. No difference between FMP or control groups was found on GI symptoms or breath H_2_ and CH_4_ in the whole cohort. A *post-hoc* analysis in patients stratified according to their fasting H_2_ levels showed that in high H_2_ producers (fasting H_2_ level≥10ppm, n = 35), FMP consumption reduced fasting H_2_ levels (p = 0.003) and H_2_ production during the challenge (p = 0.002) and tended to decrease GI discomfort (p = 0.05) vs. control product. The *Prevotella*/*Bacteroides* metabolic potential at baseline was higher in high H_2_ producers (p<0.05) vs. low H_2_ producers and FMP consumption reduced this ratio (p<0.05) vs. control product.

**Conclusions:**

The response to a fermented milk product containing *Bifidobacterium lactis* CNCM I-2494 (FMP) in patients with IBS seems to be associated with the metabolic potential of the gut microbiota.

**Trial registration:**

ClinicalTrial.gov NCT01252550.

These results were presented as congress posters at Digestive Disease Week 2016 in San Diego, USA and United European Gastroenterology Week 2016 in Vienna, Austria.

## Introduction

Gas-related symptoms such as bloating, abdominal distension and excessive flatulence are digestive complaints that can significantly affect well-being and quality of life [1–3]. Bloating and flatulence are the most common digestive symptoms reported by 15–20% of the general population in US and Europe [4, 5] and by up to 90% of patients with irritable bowel syndrome (IBS) [6]. Most patients suffering from functional gastrointestinal disorders (FGIDs) declare gas-related symptoms to be among their most bothersome symptoms [7]. No effective, safe and sustainable treatment for these symptoms is available today, partly since the physiology and pathophysiology of gas-related symptoms is complex and remains largely unknown [8, 9]. Notably, the relationship between objective markers of intestinal gas (volume, distribution, composition, frequency of evacuation) and perception of gas-related symptoms is still not clearly established [10].

Intestinal gas is produced predominantly in the colon, where unabsorbed meal residues are fermented by colonic bacteria [11, 12]. Within subjects, the volume and composition of intestinal gas production therefore vary in relation to the diet [13–15]. However, there is a great intra-, and even more so, inter-individual variability, as gas production in subjects maintained on a similar diet may differ substantially both in gas volume and composition [3, 16, 17]. This depends mainly on the composition and metabolic activity of the colonic microbiota [3]. A change in the composition of the diet, for example towards plant-based products rich in fermentable polysaccharides, can thus rapidly alter the composition and function of the gut microbiota [18, 19].

Different methods are available to assess intestinal gas production in vivo, most of them being exploratory (magnetic resonance imaging/MRI [10] or computed tomography/CT [16] and/or invasive (anal collection) [3]. The breath test is a non-invasive, standardized procedure allowing to measure gas production (H_2_, CH_4_) in end-expiratory breath samples, thereby providing an indirect assessment of intestinal gas production, as H_2_ and CH_4_ are solely produced by bacterial fermentation of undigested substrates, mainly in the colon [20]. Breath testing with measurement of H_2_ and CH_4_ after intake of different carbohydrates (e.g. lactose, fructose, glucose, lactulose) is used routinely in clinical practice to diagnose carbohydrate malabsorption, as well as small intestinal bacterial overgrowth [21]. Moreover, our group has recently developed a combined nutrient and lactulose challenge as a non-invasive test to study visceral sensitivity and to characterize symptom patterns and pathophysiology in IBS [22, 23].

Available scientific evidence based on several meta-analyses suggests that probiotics are beneficial in the management of FGIDs [24, 25]. Notably, specific probiotic strains belonging to *Bifidobacterium* have been shown to relieve the overall symptom burden in patients with FGID, and to reduce the perception of bloating and abdominal distension in patients with IBS [26]. Specifically, a fermented milk product containing *Bifidobacterium lactis* CNCM I-2494 and lactic acid bacteria (FMP) has been shown to improve well-being and digestive symptoms in women reporting minor digestive symptoms [27, 28] as well as improving bloating, digestive discomfort and reducing objectively measured abdominal distension in patients with IBS with predominant constipation (IBS-C) [29]. However, the mechanisms of action behind these clinical observations are still unclear.

The aims of the present exploratory study were i) to assess the effect of 14 days consumption of FMP on GI symptoms and exhaled H_2_ and CH_4_ during a combined nutrient and lactulose challenge test [22, 23] in patients with IBS and ii) to identify potential predictors of the intervention outcome such as intestinal gas production or pattern of symptoms at baseline.

## Materials and methods

### Study subjects

Adult patients aged between 18 and 65 years, fulfilling the Rome III criteria for IBS (all subtypes) [30] were prospectively included between May 2011 and August 2012 at a secondary/tertiary care outpatient clinic specialized in the management of FGIDs (Sahlgrenska University Hospital, Gothenburg, Sweden). The diagnosis was based on a typical clinical presentation and additional investigations if considered necessary by the gastroenterologist (MS, HT). Classification into IBS subtypes was done according to the Rome III criteria [30]. Exclusion criteria included the use of probiotics or antibiotics during the study or within one month before inclusion, severe psychiatric disease, other severe diseases, and a history of drug or alcohol abuse. All medications with known effects on the GI tract (proton pump inhibitors, laxatives, antidiarrheals, opioid analgesics, prokinetics, spasmolytics, antidepressants) were discontinued at least 48 hours before the challenge test. The study protocol was approved by the Regional Ethical Review Board in Gothenburg and all included subjects gave written informed consent. Part of the data from the subjects prior to the intervention has been presented in a recent publication [22]. However, the research question of the present publication is novel and the FMP intervention results have not been reported elsewhere.

### Study design and products

**Fig 1:**
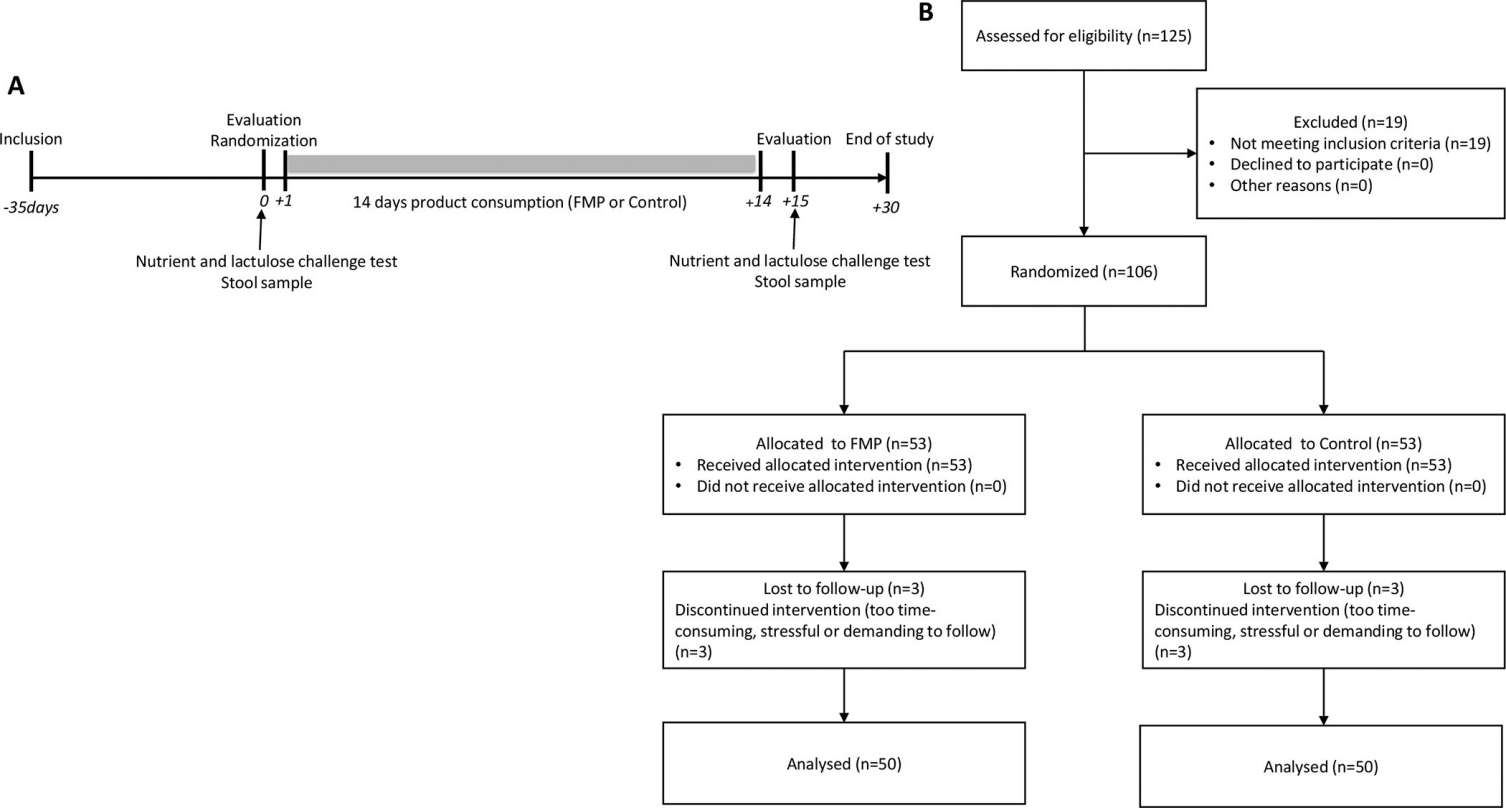
**A)** Study design **B)** Flow chart demonstrating the number of patients in the different phases of the study.

This study used a randomized, controlled, double-blind, parallel groups design (**Fig 1A**). At Visit 1 (inclusion), subjects deemed to be eligible for the study were included in a run-in period of up to 35 days designed to avoid any potential carry-over effect on gut microbiota from probiotic consumption prior to subject inclusion. At Visit 2 (baseline evaluation & randomization), subjects brought back a stool sample collected at home and performed a nutrient and lactulose challenge test in the clinic. The measured endpoints during the challenge were the intensity of eight meal-related GI symptoms, the overall level of digestive comfort and the amount of exhaled H_2_ and CH_4_ in breath (for details see supplementary material). Subjects were then randomized in a 1/1 ratio to consume 125g of either a fermented milk product (containing *Bifidobacterium animalis* subsp *lactis* CNCM I-2494, *Lactobacillus bulgaricus* CNCM I-1632 and CNCM I-1519, *Streptococcus thermophilus* CNCM I-1630 and *Lactococcus lactis* subsp *lactis* CNCM I-1631) or a control product (non-fermented milk product without bacterial strains and with similar lactose content) twice a day for 14 days. Both products were prepared at Danone Research facilities, Palaiseau, France, and shipped in blinded packaging with refrigeration to the study site at Sahlgrenska University Hospital, Gothenburg, Sweden. The randomization code was generated by an external CRO (Gothia Forum, Gothenburg, Sweden). Blinding was accomplished by ensuring that active and control products were of identical appearance, taste and texture. At Visit 3 (evaluation post 14 days product consumption period), subjects brought back a stool sample collected at home and performed a nutrient and lactulose challenge test. Compliance to the study product was measured by returning unused product to the study center. A follow-up visit (Visit 4) was performed 2 weeks after the end of the product consumption period. The randomization code was not to be broken until all assessments had been performed, all data had been entered into the database, and the database had been locked after a clean file procedure.

### Run-in period assessments

**IBS-SSS questionnaire**: the severity of IBS symptoms was determined with the validated IBS Severity Scoring System (IBS-SSS) questionnaire ranging from 0 (no symptoms) to 500 [31].

**HAD questionnaire**: general anxiety and depression were evaluated by the Hospital Anxiety and Depression scale (HAD) [32].

**PHQ 15 questionnaire**: the bothersomeness of 15 somatic symptoms was assessed by the Patient Health Questionnaire 15 (PHQ-15) [33].

**Oroanal transit time test (OATT)**: OATT was assessed in subjects by ingestion of radiopaque rings [34].

**Dietary habits**: To determine their usual intake of energy and nutrients, as well as FODMAPs (Fermentable Oligo-, Di-, Monosaccharides and Polyols), all subjects completed a 4-day food diary during the run-in period, in which the quantities consumed were entered in grams or household measures. The intake of nutrients, including FODMAPs, was calculated using a dedicated software (Dietist XP, Kostdata.se, Stockholm, Sweden).

### Fecal microbiota analysis

Fecal samples were collected before and at the end of the product consumption period from 62 IBS subjects. Fecal samples were processed in RNAlater solution (Ambion) as previously described [35]. Fecal total RNA was extracted using mechanical lysis (Fastprep FP120, ThermoSavant) followed by phenol/chloroform-based extraction as previously described [36] and analyzed by 16S sequencing on a MiSeq platform based on V3-V4 16S regions (see supporting information). The obtained data was analyzed using the open source software package Quantitative Insights Into Microbial Ecology (QIIME), v1.9 [37]. Representative sequences (i.e most abundant) for each Operational Taxonomical Units (OTUs) were taxonomically assigned using Silva database (version 119) (see supporting information). The fecal microbiota metabolic potential of a specific taxon was proportional to the number of 16S rRNA reads assigned to this taxon. The prevalence of Methanobacteriales in fecal samples was evaluated by quantitative PCR (qPCR) as described earlier [35].

### Statistical analysis

#### Clinical parameters

For analysis of parameters during the run-in period (IBS-SSS, HAD, PHQ-15, OATT, dietary habits), a one-way ANOVA was performed with intervention group (FMP, control) as unique factor for continuous variable. For categorical variables, a chi-square test was performed. For analysis of planned study endpoints assessing the effect of 14 days consumption of a fermented milk product vs. control product on GI symptoms and exhaled H_2_ and CH_4_ during a combined nutrient and lactulose challenge test, an ANCOVA model was used with intervention group (FMP, control) as only factor. For post-hoc analyses aiming to identify potential predictors of the intervention outcome based on intestinal gas production or pattern of symptoms at baseline, analyses were performed on subsets based on demonstrated differences. For this, an ANCOVA model was used with multiple factors: intervention group (FMP, control), potential predictor (eg fasting H_2_ value T0 prior to intervention) and the interaction “intervention group*predictor”. Baseline values for study endpoints were taken as covariate. If the interaction “intervention group*predictor” was significant then least square means were computed. Benjamini-Hochberg multiplicity correction was applied for all tests. The sample size in this exploratory study was not based on a power calculation. Inferential statistical tests were performed with alpha risk level at 5%. Univariate and multivariate statistical analyses were performed using JMP v11 software (SAS Institute Inc., Cary, NC) and R (version 3.1.2).

#### Microbiota parameters

We first assessed the impact of intervention on gut microbiota in the whole cohort, followed by analysis on subgroups as defined by the post-hoc stratification. DESeq2, an approach specifically designed for RNA sequencing analysis and suitable for low number of subjects [38] was used for statistical analysis of microbiota parameters. Full statistical methods on microbiota parameters can be found in **supporting information**.

## Results

### Clinical characteristics at baseline

Clinical characteristics at baseline are demonstrated in **Table 1**. No differences were found for age, gender, Rome III subtype, BMI, IBS-SSS or PhQ-15 scores between groups randomized to FMP and control product, but the scores for HAD anxiety and depression were higher in patients randomized to receive the control product.

**Table 1:** Clinical characteristics of randomized subjects.

Mean (SD)	FMP (n = 53)	Control (n = 53)	*P* value
**age**	35.3 (11.5)	35.7 (10.6)	NS
**gender F/M (%)**	64.2 / 35.8	56.6 / 43.4	NS
**BMI**	23.4 (3.2)	23.2 (4.0)	NS
**IBS-SSS**	292.1 (110.3)	268.3 (99.0)	NS
**HAD anxiety**	6.9 (3.9)	9.0 (4.7)	*
**HAD depression**	4.0 (2.6)	5.9 (3.5)	*
**PHQ-15**	12.6 (5.7)	13.1 (4.5)	NS
**IBS-C, n (%)**	10 (18.9%)	10 (18.9%)	NS
**IBS-D, n (%)**	17 (32.1%)	16 (30.2%)	NS
**IBS-M, n (%)**	21 (39.6%)	16 (30.2%)	NS
**IBS-U, n (%)**	5 (9.4%)	11 (20.8%)	NS

SD: Standard Deviation; BMI: body mass index; IBS-SSS: IBS Severity Scoring System; HAD: hospital anxiety and depression scale; PHQ-15: patient health questionnaire 15; IBS-C: irritable bowel syndrome with constipation; IBS-D: irritable bowel syndrome with diarrhea; IBS-M: irritable bowel syndrome with mixed pattern; IBS-U: irritable bowel syndrome unsubtyped; all questionnaire data are expressed as mean total scores; Statistical significance is determined by Oneway ANOVA. Benjamini Hochberg multiplicity correction was applied. NS for p value >0.05

* <0.05

#### Effect of intervention on planned study endpoints: GI symptoms, overall digestive comfort and exhaled H_2_ and CH_4_

Out of 125 subjects assessed for eligibility, a total of 106 patients were randomized and 100 patients completed the study (**Fig 1B**). 19 subjects did not meet the study inclusion criteria and 6 subjects (3 per group) discontinued the study. No significant difference between patients who received FMP or control product were found on GI symptoms, overall digestive comfort and exhaled H_2_ and CH_4_ following the 14 days intervention. All results on the planned study endpoints are presented in **Table 2**.

**Table 2:** Intervention results on planned study endpoints.

Mean (SD)	FMP (n = 50)	Control (n = 50)	*P* value
**Δ T0 H_2 ppm_**	-3.50 (16.00)	4.70 (15.50)	0.23
**Δ T0 CH_4 ppm_**	1.00 (9.30)	-0.20 (5.00)	0.51
**Δ H_2 ppm_**	0.57 (19.10)	8.10 (25.14)	0.23
**Δ CH_4 ppm_**	1.20 (10.39)	0.24 (9.02)	0.54
**Δ gas**	-0.23 (3.11)	-0.65 (2.81)	0.52
**Δ bloating**	-0.42 (3.07)	-0.77 (2.88)	0.54
**Δ discomfort**	-0.87 (2.85)	-1.08 (2.83)	0.54
**Δ distension**	-0.54 (2.60)	-0.92 (2.86)	0.54
**Δ nausea**	-0.35 (3.36)	-0.22 (2.78)	0.79
**Δ rumbling**	-0.22 (2.81)	-0.88 (2.94)	0.24
**Δ urgency**	0.01 (2.82)	-0.37 (2.60)	0.39
**Δ pain**	0.01 (2.84)	-0.66 (2.34)	0.23
**Δ comfort**	-0.20 (2.60)	0.70 (2.40)	0.24

SD: Standard Deviation; FMP: fermented milk product (FMP); Control: non-fermented milk product; ppm: parts per million; H_2_: hydrogen; CH_4_: methane; T0: fasting value; all statistical analyses are covariance analyses of the 4h mean change (Δ) on measured endpoints from 1^st^ to 2^nd^ nutrient-lactulose challenge between the two study arms adjusted for 1^st^ challenge values; all significance tests were two-sided and conducted at the 5% significance level; Benjamini Hochberg multiplicity correction was applied.

### *Post-hoc* stratification of patients and effect of intervention

**Fig 2:**
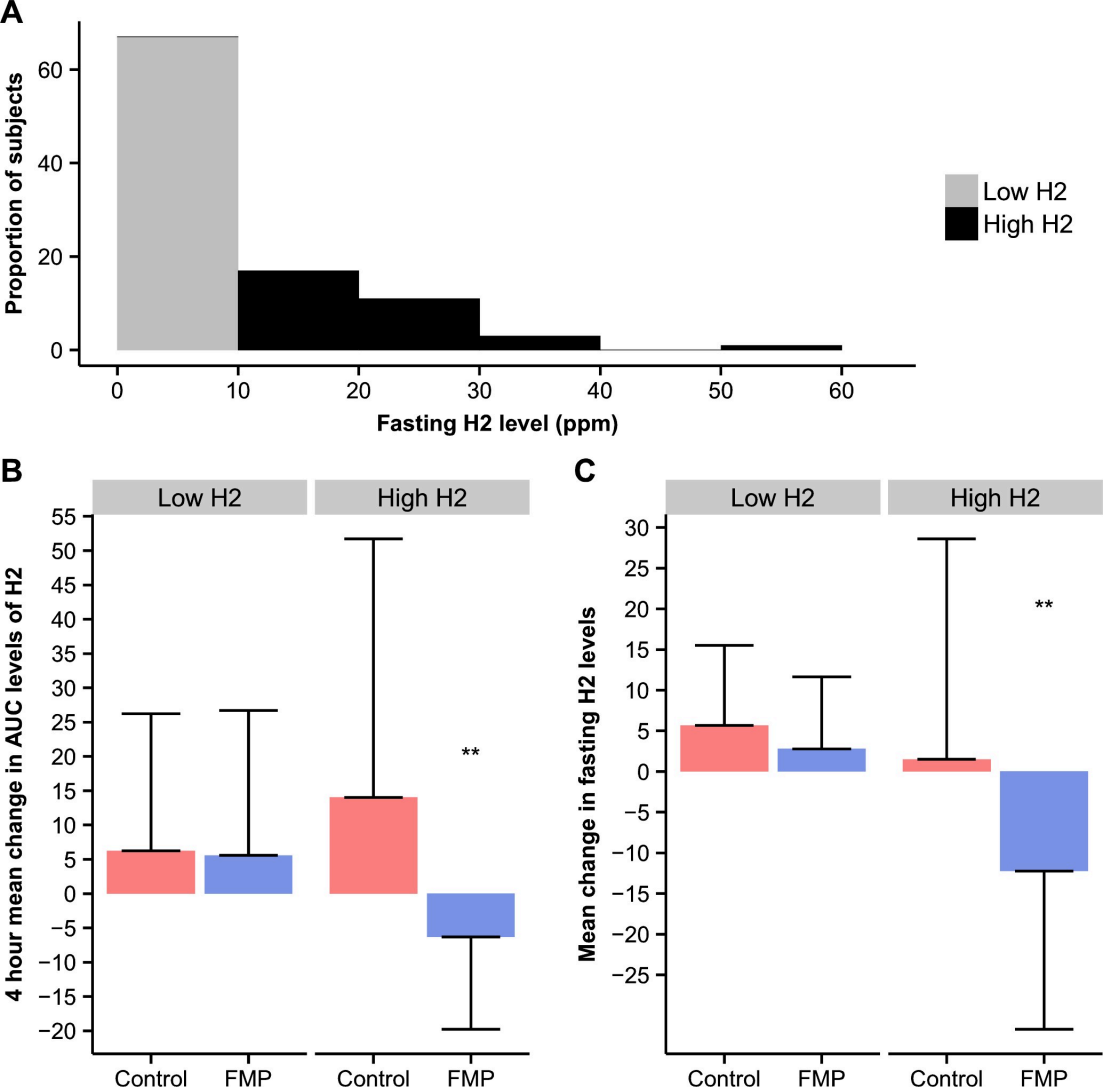
Effect of intervention on breath hydrogen in IBS patients stratified according to fasting H_2_ levels. **A)** Distribution of subjects in % according to H_2_ fasting value **B)** Effect of the intervention on the 4h mean change in AUC levels of H_2_ **C)** Effect of the intervention on the mean change in fasting H_2_ levels (T0); FMP: fermented milk product; Control: non-fermented milk product; High H_2_: fasting H_2_ level≥10ppm (n = 33); Low H_2_: fasting H_2_ level<10ppm (n = 67); ppm: parts per million; H_2_: hydrogen; T0: fasting value; statistical analyses for **B)** and **C)** are covariance analyses of the mean change (Δ) of the measured endpoints from 1^st^ to 2^nd^ nutrient-lactulose challenge between the two study arms adjusted for first challenge values; all significance tests were two-sided and conducted at the 5% significance level. Interaction was evaluated at 10% significance level; NS for p value >0.05, * <0.05, ** <0.01, *** <0.001.

Most subjects (67%) had fasting H_2_ levels lower than 10ppm prior to intervention, with nearly half of the subjects (48%) having values between 0 and 2ppm (**Fig 2A**). A binary classification with an arbitrary cut-off at 10ppm H_2_ was performed prior to intervention, separating subjects into “low fasting H_2_ producers” (n = 67) and “high fasting H_2_ producers” (n = 33). No significant differences were found for age, gender, BMI, IBS subtype distribution, OATT, intake of energy or nutrients including FODMAPs, total scores for IBS-SSS, PHQ-15, HAD anxiety or HAD depression between high and low H_2_ producers. All data are summarized in **Table 3**. A *post-hoc* analysis on study endpoints (GI symptoms, overall digestive comfort and exhaled H_2_ and CH_4_) was conducted in patients stratified according to their fasting H_2_ levels (T0) prior to intervention. The FMP intervention in high H_2_ producers reduced the 4h mean AUC of H_2_ during the challenge vs. control (p = 0.002) (**Fig 2B**), but not CH_4_ (p = 0.31), and tended to decrease discomfort during the challenge vs. control (p = 0.05). The FMP intervention also reduced mean fasting levels (T0) of H_2_ vs. control (p = 0.004) in high H_2_ producers (**Fig 2C**). In contrast, no effect of the FMP intervention was seen on exhaled gas in low H_2_ producers, even if CH_4_ tended to be higher in the FMP group (p = 0.08). Regarding the evolution of GI symptoms during the challenge in low H_2_ producers, a difference was observed between the FMP and control groups for gas, discomfort, rumbling and pain, with a greater reduction of these symptoms in the control group. All post-hoc intervention results are compiled in **Table 4**.

**Table 3:** Clinical characteristics of fasting H_2_ based subgroups (*post-hoc*).

Mean (SD)	Low H_2_ (n = 71)	High H_2_ (n = 35)	*P* value
**age**	36.0 (10.5)	34.5 (12.1)	0.67
**gender F/M (%)**	64.8 / 35.2	51.4 / 48.6	0.19
**BMI**	23. 4 (3.9)	23.1 (3.0)	0.67
**IBS-SSS**	281.2 (100.0)	278.1 (116.0)	0.90
**HAD anxiety**	8.3 (4.5)	7.4 (4.3)	0.56
**HAD depression**	5.4 (3.4)	4.1 (2.5)	0.44
**PHQ-15**	13.2 (4.9)	12.2 (5.5)	0.56
**IBS-C, n (%)**	15 (21.1)	5 (14.3)	0.83
**IBS-D, n (%)**	21 (29.6)	12 (34.3)	
**IBS-M, n (%)**	25 (35.2)	12 (34.3)	
**IBS-U, n (%)**	10 (14.1)	6 (17.1)	
**OATT (days)**	1.5 (1.2)	1.3 (0.8)	0.56
**Energy from diet (kcal)**	2198.7 (598.7)	2037.0 (554.0)	0.56
**%Carbohydrates**	44.2 (8.5)	45.2 (8.3)	0.67
**%Protein**	16.7(3.6)	17.2 (4.4)	0.67
**%Fat**	36.1 (6.8)	34.4 (7.9)	0.60
**FODMAPs (g)**	16.3 (10.0)	14.1 (8.3)	0.56

SD: Standard Deviation; High H_2_: fasting H_2_ level≥10ppm; Low H_2_: fasting H_2_ level<10ppm; BMI: body mass index; IBS-SSS: IBS Severity Scoring System; HAD: hospital anxiety and depression scale; PHQ-15: patient health questionnaire 15; IBS-C: irritable bowel syndrome with constipation; IBS-D: irritable bowel syndrome with diarrhea; IBS-M: irritable bowel syndrome with mixed pattern; IBS-U: irritable bowel syndrome unsubtyped; OATT: oroanal transit time; FODMAP: fermentable, oligo-, di-, mono-saccharides and polyols; all questionnaires are expressed as mean total scores; Statistical significance is determined by Oneway ANOVA for quantitative parameters. Multiple testing strategy consisted in Benjamini Hochberg adjustment for quantitative parameters and two-sided Chi^2^ test for qualitative parameters.

**Table 4:** Intervention results in fasting H_2_ based subgroups (*post-hoc*).

		Low H_2_ (n = 67)			High H_2_ (n = 33)		
mean (SD)	Interaction P-value	FMP (n = 29)	Control (n = 38)	*P* value	FMP (n = 21)	Control (n = 12)	*P* value
**Δ T0 H_2 ppm_**	0.06	2.8 (8.9)	5.7 (9.9)	0.31	-12.2 (19.5)	1.5 (27.1)	0.004**
**Δ T0 CH_4 ppm_**	0.36	3.2 (6.2)	0.3 (4.5)	NA	-2.1 (11.9)	-1.5 (6.4)	NA
**Δ H_2 ppm_**	0.01*	5.6 (21.1)	6.2 (20)	0.79	-6.3 (13.5)	14 (37.7)	0.002**
**Δ CH_4 ppm_**	0.07	3.9 (7.0)	-0.2 (8.1)	NA	-2.5 (13.1)	1.7 (11.8)	NA
**Δ gas**	0.03*	0.6 (2.4)	-1.0 (3.1)	0.04*	-1.4 (3.7)	0.4 (1.0)	0.20
**Δ bloating**	0.18	0.0 (2.7)	-1.0 (3.0)	NA	-1.0 (3.5)	0.1 (2.3)	NA
**Δ discomfort**	0.005**	-0.1 (2.6)	-1.5 (2.9)	0.03*	-1.9 (2.9)	0.1 (2.2)	0.05
**Δ distension**	0.20	-0.1 (2.4)	-1.0 (3.2)	NA	-1.1 (2.8)	-0.5 (1.1)	NA
**Δ nausea**	0.20	-0.3 (2.9)	-0.6 (2.7)	NA	-0.5 (4.0)	0.9 (3.0)	NA
**Δ rumbling**	0.01*	0.6 (2.9)	-1.2 (3.0)	0.003**	-1.4 (2.3)	0.1 (2.7)	0.34
**Δ urgency**	0.10	0.4 (2.8)	-0.7 (2.6)	NA	-0.5 (2.9)	0.6 (2.4)	NA
**Δ pain**	0.02*	0.3 (3.2)	-1.0 (2.2)	0.004**	-0.4 (2.3)	0.4 (2.5)	0.43
**Δ comfort**	0.93	0.1 (2.1)	0.9 (2.7)	NA	-0.7 (3.1)	0.2 (1.4)	NA

SD: Standard Deviation; FMP: fermented milk product (FMP); Control: non-fermented milk product; High H_2_: fasting H_2_ level≥10ppm; Low H_2_: fasting H_2_ level<10ppm; ppm: parts per million; H_2_: hydrogen; CH_4_: methane; N/A: not applicable; T0: fasting value; all statistical analyses are covariance analyses of the 4h mean change (Δ) on measured endpoints from 1^st^ to 2^nd^ nutrient-lactulose challenge between the two study arms adjusted for 1^st^ challenge values; all significance tests were two-sided and conducted at the 5% significance level. Interaction was evaluated at 10% significance level

* <0.05

** <0.01

### Effect of the intervention on gut microbiota metabolic potential

**Fig 3:**
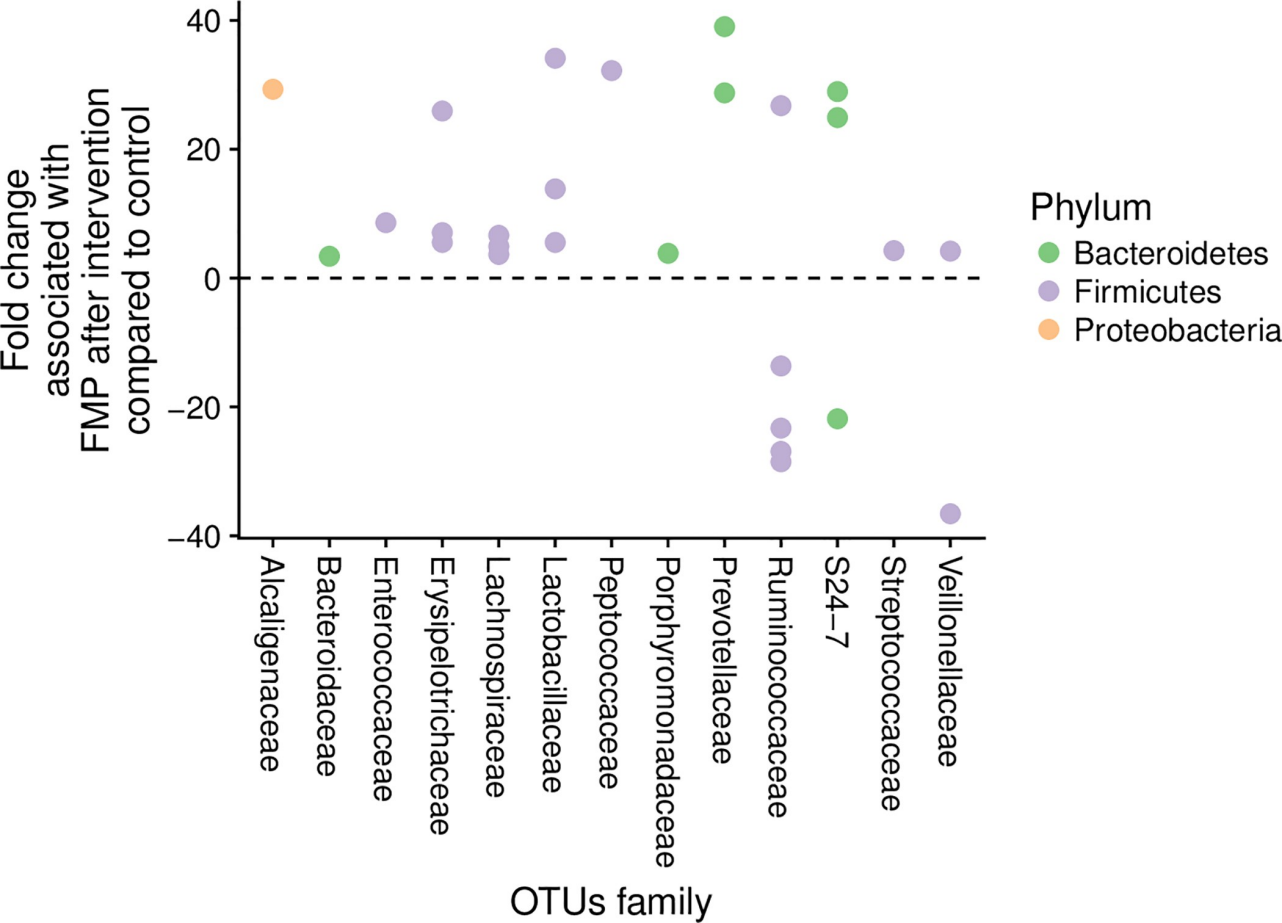
Impact of the intervention on the metabolic potential of the gut microbiota in subjects consuming FMP (n = 32) or control product (n = 30). Effect of the intervention on microbial OTUs. Each dot corresponds to an OTU colored according to their taxonomical affiliation (Phylum).

We examined the effect of the intervention on the metabolic potential of the gut microbiota. Six genera were identified as dominant and these included *Bacteroides*, *Prevotella*, Ruminococcaceae *Incertae Sedis*, *Blautia*, *Faecalibacterium* and *Bifidobacterium*. Together, the relative abundance of these six genera represented on average 40% of the microbiota metabolic potential in this study. None of these genera were different before as compared to after intervention (p>0.05). Next, we examined the response of the gut microbiota at a lower taxonomical level, i.e. OTUs. OTUs that were altered by FMP represented a median of 7.1% of 16S rRNA sequences. Fourteen (14) out of 21 OTUs that were up-regulated by FMP belonged to the Firmicutes phylum (**Fig 3**).

#### Metabolic potential of gut microbiota in fasting H_2_-based subgroups and effect of the intervention

**Fig 4:**
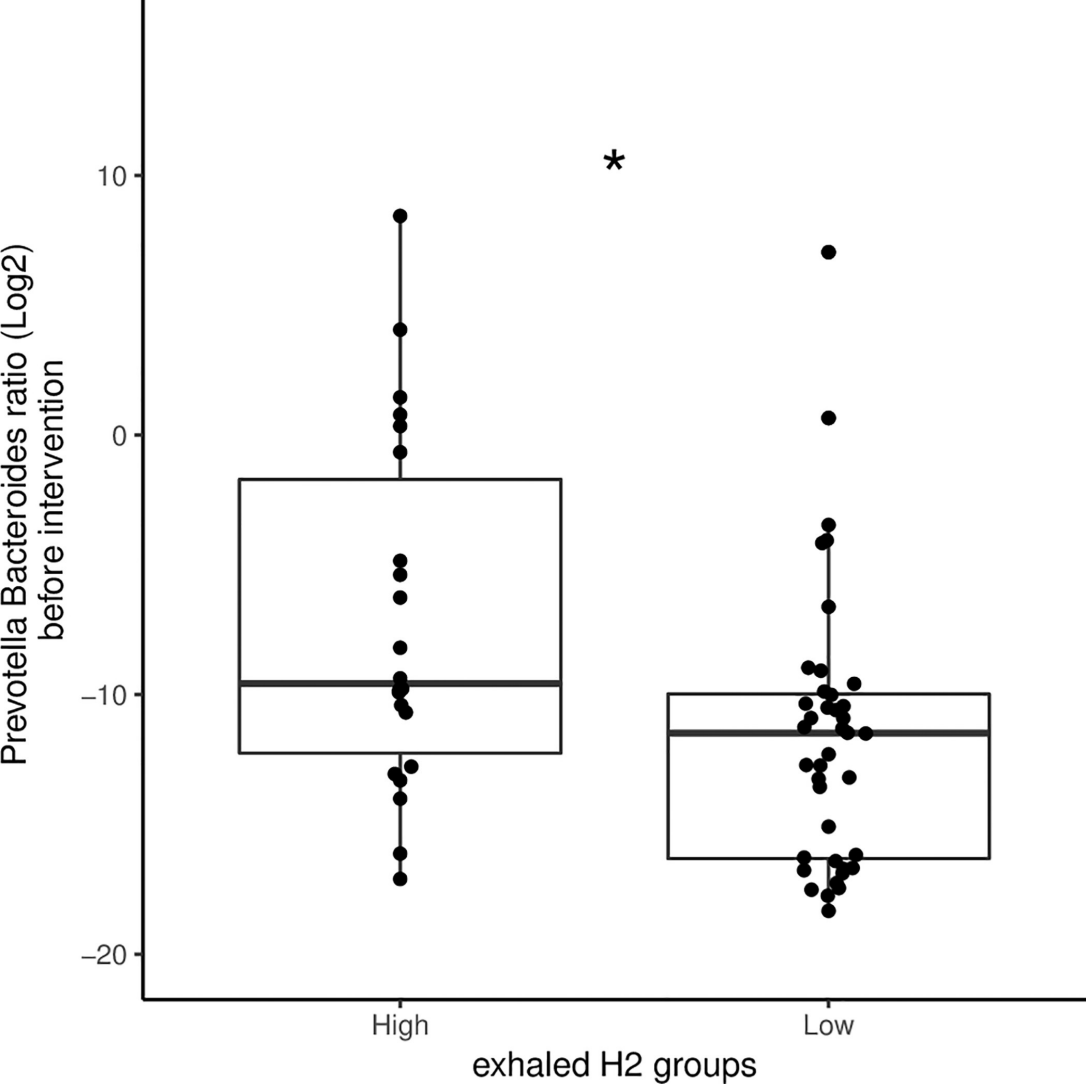
Gut microbiota of IBS patients according to their fasting H_2_ levels. Metabolic potential of the *Prevotella/Bacteroides* ratio in low (n = 40) and high (n = 22) H_2_ producers; Statistical significance was determined by Wilcoxon test; *P < 0.05.

**Fig 5:**
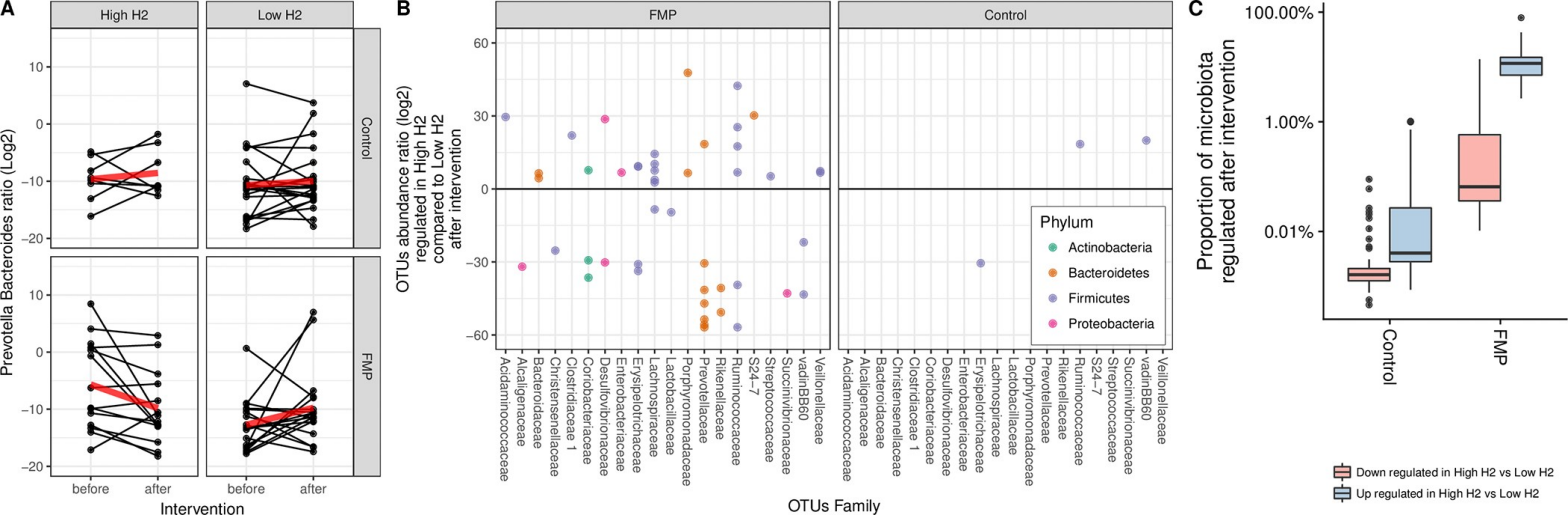
Response of gut microbiota to intervention according to fasting H_2_ levels in IBS patients. **A)** Metabolic potential of the *Prevotella* / *Bacteroides* ratio. Each line represents a subject before and after intervention. The red line represents the median evolution. **B)** Effect of the intervention (FMP vs. control) on microbial OTUs in high and low H_2_ producers. Each dot corresponds to an OTU with its associated family depicted on the x axis. OTUs are colored according to their taxonomical affiliation (Phylum). **C)** Proportion (showed in %) of whole microbiota metabolic potential regulated in high H_2_ versus low H_2_ producers upon intervention. High H_2_/control n = 8; High H_2_/FMP n = 14; Low H_2_/control n = 22; Low H_2_ FMP n = 18.

Next, we evaluated whether the dominant active microbiota prior to intervention, measured by 16S rRNA sequencing, differed at the genus level between high (n = 22) and low H_2_ producers (n = 40) for which fecal samples could be analyzed. From the six dominant genera, *Prevotella* and *Bacteroides* were significantly different between high and low H_2_ producers (p<0.05, FDR q-value <0.2). The ratio of *Prevotella* / *Bacteroides* was higher in high H_2_ compared to low H_2_ producers prior to intervention (p<0.05) (**Fig 4**). In addition, a targeted analysis on known H_2_-consuming bacteria was performed. The prevalence of Methanobacteriales, evaluated by quantitative PCR, abundance of *Desulfovibrio* (sulphate-reducing bacteria) and Blautia (acetogen) did not differ between H_2_-based subgroups (**S1 Fig**). Within the high H_2_ producers, there was a reduction in the *Prevotella / Bacteroides* ratio following 14 days consumption of the FMP (p<0.05), which was not observed after the control product (**Fig 5A**). DESeq2 analysis showed that 42 OTUs belonging to a number of families within Firmicutes, Bacteroidetes and Proteobacteria responded differently in high vs. low H_2_ producers after FMP, while only 3 OTUs were altered after the control product (**Figs 5B and S2**). Seven OTUs belonged to Prevotellaceae family and six of them were downregulated after FMP in high H_2_ producers vs. low H_2_ producers. In addition, the most dominant active OTUs on average, which belonged to Bacteroidaceae, was upregulated in high H_2_ producers after FMP vs. low H_2_ producers. The post-hoc stratification of patients according to fasting H_2_ levels showed that consumption of FMP significantly altered OTUs (p<0.05) representing on average 11.6% of the total 16S rRNA sequence vs. 7.1% in the whole cohort (**Fig 5C**). The prevalence of known H_2_-consuming bacteria (Methanobacteriales, *Desulfovibrio*, Blautia) did not change in any group after the intervention (**S1 Fig**).

## Discussion

In this exploratory study, we aimed i) to assess the effect of 14 days consumption of a FMP on GI symptoms and exhaled H_2_ and CH_4_ during a combined nutrient and lactulose challenge test in patients with IBS and ii) to identify potential predictors of the intervention outcome, such as intestinal gas production or pattern of symptoms at baseline.

No effect of the intervention was seen in the whole cohort. However, using *post-hoc* analyses, we demonstrated that a FMP intervention can reduce the production of intestinal gas in a subgroup of IBS patients. Hence, individuals exhibiting higher fasting production of hydrogen prior to the intervention benefited the most from the FMP. These subjects were characterized by a distinct gut microbiota activity level signature but did not differ otherwise from other patients regarding clinical parameters or dietary intake. These findings suggest that fasting breath H_2_ testing may be a potential predictor of FMP intervention outcome.

In the present trial, no difference between the FMP and the control intervention were found on study endpoints when considering the whole cohort. Patients with IBS form a highly heterogeneous population, with numerous subgroups identified based on severity and pattern of symptoms and the underlying pathophysiology [35, 39, 40]. This represents a true challenge when evaluating the efficacy of new treatments in this population. In this exploratory study, we chose to include all patients with IBS regardless of their symptom severity or predominant bowel habits. Our objective was to identify potential predictors of the intervention outcome within this heterogeneous cohort. This approach may help to recruit more specific and homogeneous cohorts of patients in future studies based on a surrogate marker of gut microbiota activity, thereby enhancing the chances of success.

Several randomized controlled trials have been conducted with probiotics in patients with FGID, and particularly in IBS [41–43]. To the best of our knowledge, none of these studies have shown an impact on intestinal gas production, even in subgroups of patients. We previously showed that the intensity of GI symptoms elicited by a nutrient and lactulose challenge in patients with IBS correlates poorly with levels of H_2_ and CH_4_ in breath [22, 23]. This was also the case in the present study. However, an interesting finding was a tendency towards reduction of discomfort during the challenge test following consumption of FMP in the subgroup of high H_2_ producing IBS patients. In these patients, perception of symptoms could in part be linked to objectively increased volumes of intestinal gas, and the FMP intervention would help to relieve the patients from their symptoms by decreasing the amount of gas. Previous attempts to use the lactulose hydrogen breath test (LHBT) as a predictor for dietary intervention outcomes in patients with FGID have failed [44]. However, in contrast to our study where the fasting H_2_ value was used to stratify patients prior to intervention, previous attempts used either AUC or threshold values for H_2_ following LHBT [44]. For a given individual, the reproducibility of the fasting H_2_ value over time is most certainly influenced by diet among other factors. A diet rich in fermentable carbohydrates leads to higher production of intestinal gas [3], but by standardizing conditions the day prior to the test, for example by providing a standard diet and controlling the duration of fasting, a better reproducibility in the measurements of fasting H_2_ might be achieved. A test-retest methodological study would be needed to validate this assumption.

There are several potential mechanisms of action for the FMP based on the post-hoc analyses on exhaled gas in our study. The first hypothesis on how consumption of FMP may reduce exhaled H_2_ is via modulation of the gut microbiota activity. The response of the gut microbiota to consumption of FMP has been reported in several clinical trials in healthy subjects and IBS patients [45, 46]. While these studies did not show an overall change of gut microbiota composition using 16S approach and metagenomics, McNulty *et al*. [45] reported that 7-week consumption of FMP in healthy women induced changes in the activity of some metabolic pathways related to carbohydrates and short chain fatty acids. In line with this, in the present study, we hypothesize that the differences observed in exhaled H_2_ between individuals could be related to the gut microbiota metabolic potential. While no difference could be observed in dominant genera, a higher metabolic potential of the *Prevotella/Bacteroides* ratio was observed in high H_2_ producers as compared to low H_2_ producers prior to the intervention. While the value of these findings is not yet fully understood, metagenomics analysis have shown that both genera exhibit different genetic capacity to produce hydrogenases [47]. FMP consumption also influenced the gut microbiota activity to a larger extent than the control product, especially in high H_2_ producers. In these patients, a higher number of OTUs were modulated by FMP vs. low H_2_ producers. However, the prevalence of Methanobacteriales able to consume H_2,_ and abundance of genera amongst sulphate-reducing and acetogenic bacteria did not differ between low and high H_2_ producers at baseline or after the intervention. Lastly, we can hypothesize that the metabolism of the candidate probiotic strain *B*. *animalis* subsp *lactis*, previously shown to be active in the gut microbiota [45, 46], may reduce H_2_ production during the nutrient and lactulose challenge by metabolizing lactulose. Such metabolism would reduce the availability of lactulose to colonic bacteria that can degrade lactulose and produce gas [48]. However, FMP was not consumed close in time to the challenge test, as the participants arrived in the morning to the laboratory after an overnight fast.

We acknowledge that there are limitations with our study. First, the stratification between high and low H_2_ producers was not planned *a priori* in the study protocol and has been performed as a *post-hoc* analysis. Therefore, sample size in the FMP and control groups is unbalanced, with nearly twice as many individuals in the group who consumed FMP relative to the group who consumed the control product. Moreover, stratification of patients has been performed based on a single value, i.e. the fasting H_2_ value prior to the intervention. Reproducibility of this measurement, and therefore robustness of the ratio between high and low H_2_ producers needs to be assessed in an independent study. It should also be mentioned that 16S rRNA sequencing, although being a useful tool to assess the metabolic potential of gut microbiota, cannot provide information about functionality. To better understand the overall picture, techniques like metatranscriptomics would be needed. Finally, all patients were included at a secondary/tertiary referral center, which could prevent extrapolation of the results to an IBS population at the societal level. Therefore, the present findings need to be confirmed in a study where the subjects would be randomized according to their fasting H_2_ levels and performed with subjects from the general population.

In conclusion, the present findings indicate that 14 days consumption of a fermented milk product containing *B*. *lactis* CNCM I-2494 and lactic acid bacteria can reduce the production of intestinal gas in a subgroup of patients who are high H_2_ producers, both in fasting conditions and following a nutrient and lactulose challenge. Fasting H_2_ levels and *Prevotella/Bacteroides* ratio metabolic potential were associated with the outcome of the intervention. These findings need to be confirmed to assess whether the metabolic potential of gut microbiota can predict the response to a FMP in patients with IBS.

## Supporting information

S1 Fig Prevalence of Methanobacteriales, *Blautia* and *Desulfovibrio* detected in fecal sample. Methanobacteriales were detected in fecal DNA by qPCR **A)** at baseline in Low (n = 54) and High (n = 24) H_2_ producers and **B)** stratified by study group, before and after intervention. *Blautia* and *Desulfovibrio* 16S RNA-seq reads abundance were depicted into tertiles. Low, medium and high corresponded respectively to the first, second and third tertiles. Subject prevalence for each tertile was indicated at baseline in respectively **C) E)** and stratified by study group, before and after intervention respectively in **D) F)**; High H_2_ control (n = 9), Low H_2_ control (n = 29), High H_2_ FMP (n = 15), Low H_2_ FMP (n = 25).
(TIFF)

S2 Fig Differentially abundant OTUs and their family identified using DESeq2. Samples were compared between high H_2_ (n = 24) and low H_2_ (n = 54) producers after intervention.
(TIFF)

S1 File Supporting materials and methods. (DOCX)

S2 File MOSAIC clinical study protocol. (PDF)

S3 File CONSORT 2010 checklist. (DOC)
