# Supplementary figures and images for: Fasting breath H2 and gut microbiota metabolic potential are associated with the response to a fermented milk product in irritable bowel syndrome
Source: PLoS One. 2019 Apr 4;14(4):e0214273. doi: 10.1371/journal.pone.0214273 (PMC6448848; doi:10.1371/journal.pone.0214273)

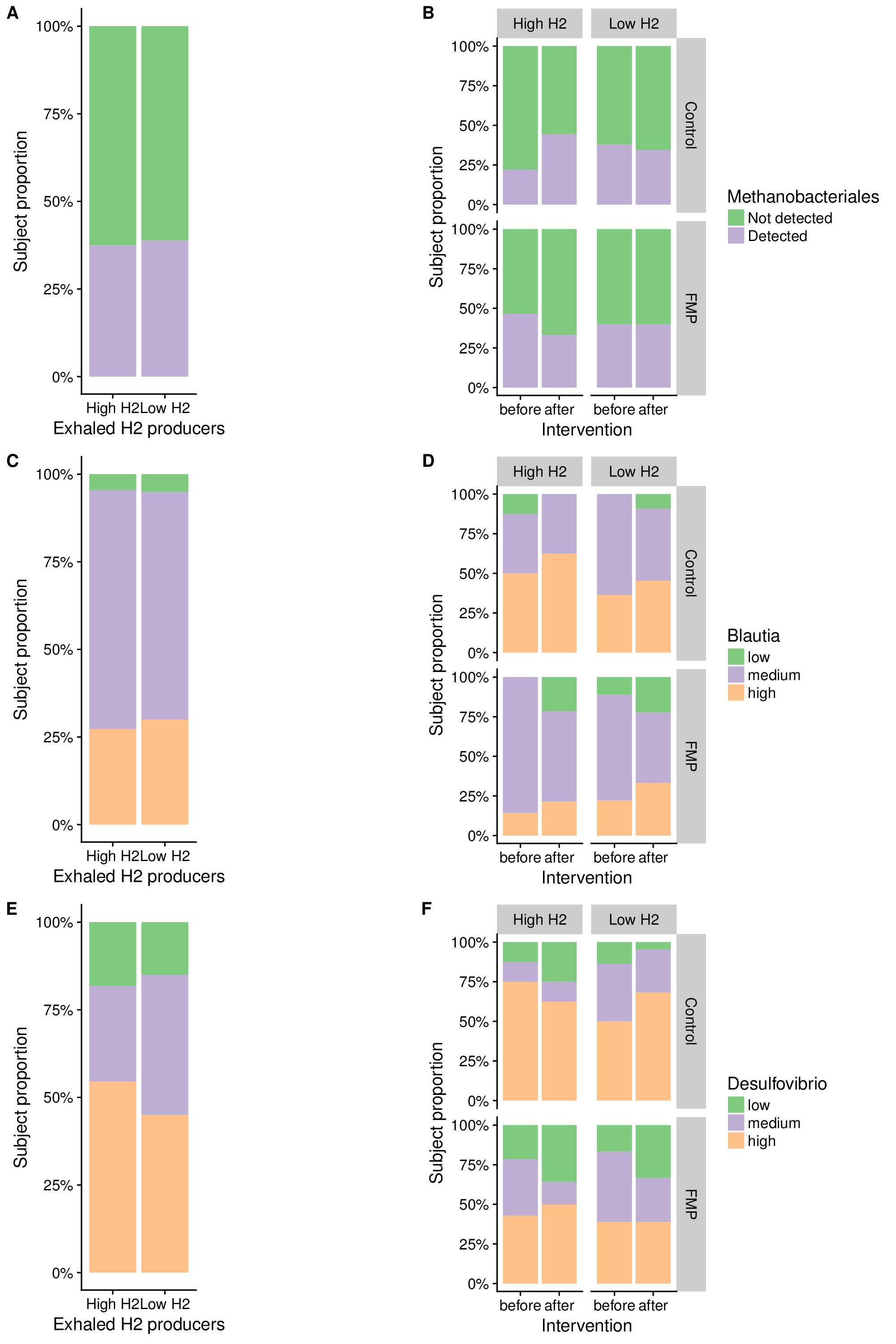

Supplement: S1 Fig — Methanobacteriales were detected in fecal DNA by qPCR A) at baseline in Low (n = 54) and High (n = 24) H2 producers and B) stratified by study group, before and after intervention. Blautia and Desulfovibrio 16S RNA-seq reads abundance were depicted into tertiles. Low, medium and high corresponded respectively to the first, second and third tertiles. Subject prevalence for each tertile was indicated at baseline in respectively C) E) and stratified by study group, before and after intervention respectively in D) F); High H2 control (n = 9), Low H2 control (n = 29), High H2 FMP (n = 15), Low H2 FMP (n = 25). (TIFF) [file pone.0214273.s001.tiff]

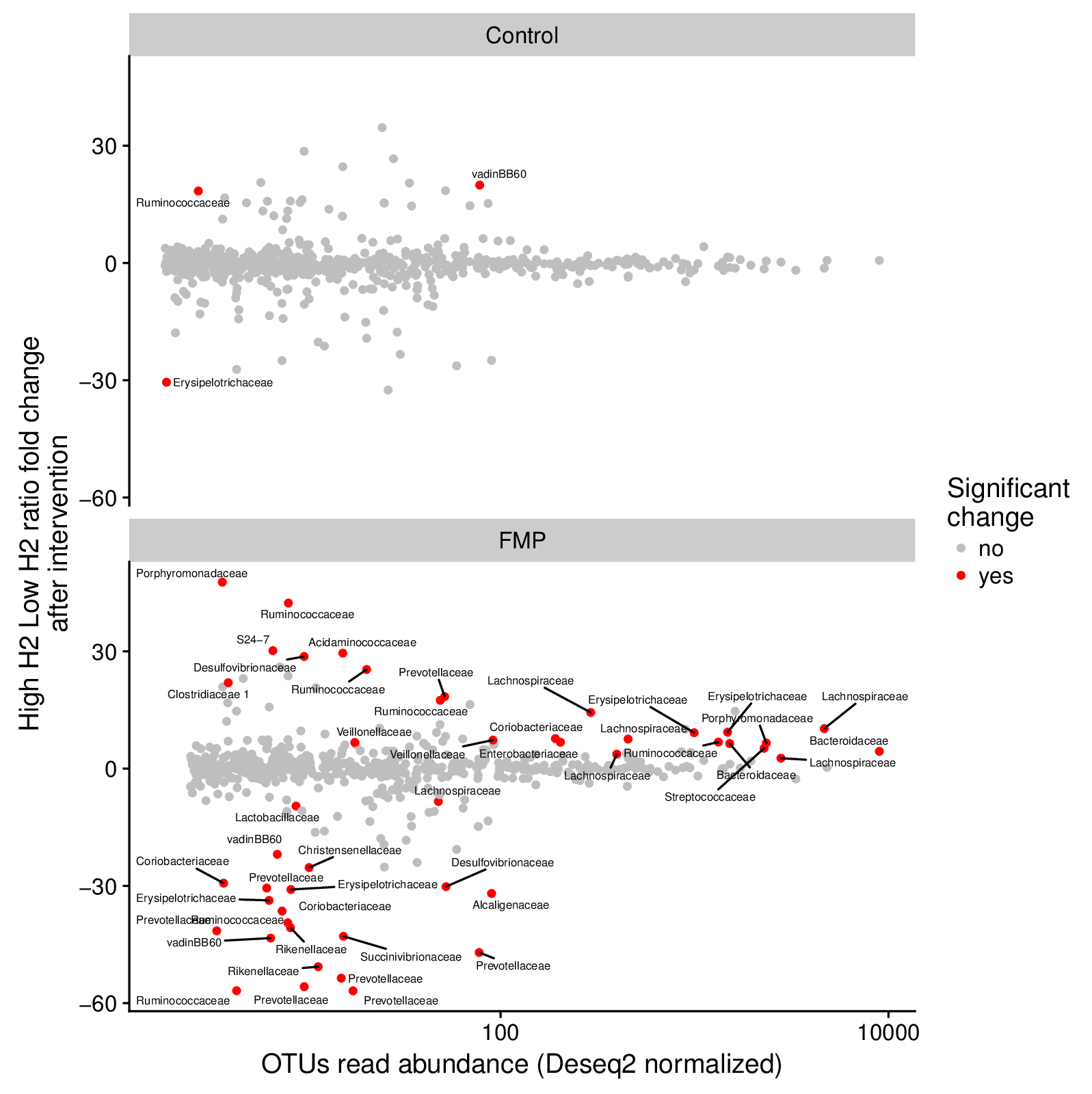

Supplement: S2 Fig — Samples were compared between high H2 (n = 24) and low H2 (n = 54) producers after intervention. (TIFF) [file pone.0214273.s002.tiff]
