# Supplementary material for: Fasting breath H2 and gut microbiota metabolic potential are associated with the response to a fermented milk product in irritable bowel syndrome
Source: PLoS One. 2019 Apr 4;14(4):e0214273. doi: 10.1371/journal.pone.0214273 (PMC6448848; doi:10.1371/journal.pone.0214273)
Supplement: S1 File — (DOCX) [file pone.0214273.s003.docx]

**Supporting information**

**Supporting materials and methods**

**Nutrient and lactulose challenge test**

This challenge has already been described in detail previously [1, 2]. Briefly, after the subject had been instructed to eat a low fibre diet the day before the test, all participants arrived to the laboratory at 7:30 a.m. after an overnight fast. The combined nutrient and lactulose challenge test (400ml Nutridrink®, 1.5 kcal/ml, 16% proteins, 49% carbohydrates, 35% fat, gluten free, lactose<0.025g/100ml and 25g of lactulose, Meda, Sweden) was served at 8:00 a.m. The severity of eight GI symptoms rated on a scale from 0-20 (ranging from no symptoms to the worst conceivable symptoms: gas, bloating, abdominal discomfort, abdominal distension, nausea, stomach rumbling, urgency to have a bowel movement, abdominal pain), the overall digestive comfort rated on a scale from 0-20 (ranging from extremely uncomfortable to extremely comfortable) and the amount of exhaled H_2_ and CH_4_ in breath were all assessed every 15 min starting at T0 (fasting value) before the test meal and then during 4h following meal intake (expressed as 4h mean area under the curve for H_2_, CH_4_ and symptoms). End-expiratory breath samples were collected in a system used for the sampling and storing of alveolar air (GaSampler system, QuinTron Instrument Company, Milwaukee, WI, USA) and analyzed immediately using a gas chromatograph (QuinTron Breath Tracker, QuinTron Instrument Company, Milwaukee, WI, USA). Exhaled H_2_ and CH_4_ were measured in parts per million (ppm). The challenge test was performed twice, prior to the intervention and at the end of the 14 days product consumption period.

**Processing of microbiota samples**

Fecal RNA was cleaned twice with Cleanup RNeasy Minielute kit (Qiagen) using QIAvac Connecting System (Qiagen). Final quality control was performing with Agilent RNA 6000 Nano and RNA 6000 Pico Kits in an Agilent 2100 Bioanalyzer. First-strand cDNA synthesis using SuperScript II was performed following manufacturer instructions (Invitrogen) and second-strand synthesis was done with 200 μM of each of the four deoxynucleoside triphosphates, 1 μM of reverse primer, 5 U of FastStart HiFi Polymerase, and the appropriate buffer with MgCl_2_ supplied by the manufacturer (Roche, Mannheim, Germany). Thermal cycling consisted of initial denaturation at 94°C for 5 minutes followed by 1 cycle of annealing at 50°C for 50 min, and extension at 72°C for 10 min. Then the protocol for 16s rRNA library by Illumina was followed in second PCR step. Amplification was performed using primers targeting V3-V4 regions of the 16S rRNA (341 F: CCTACGGGNGGCWGCAG 785R GACTACHVGGGTATCTAATCC) [3]. The samples were loaded in several flowcells in the MiSeq Platform from Illumina using a 300PE combination following manufacturer specifications. Analyses were performed using QIIME v.1.9. After quality filtering, an average of 70 000 ± 30 000 sequences per sample were included for downstream analyses. Reads were clustered into Operational Taxonomic Units (OTUs) defined at 97% identity using Vsearch [4] and representative sequences for each OTU were aligned and taxonomically assigned using Silva database (version 119).

**Statistical analysis of microbiota parameters**

Relative abundance of microbial genera and OTUs was collected from the Qiime biom file for each fecal sample. Microbial genera were considered as dominant if their relative abundance was higher than 20% in at least one sample. For each genera, a non-parametric test (Wilcoxon) was used to compare samples between intervention groups (FMP, control) and post-hoc stratification. In case of test multiplicity, false discovery rate (FDR q-value) was computed. Genera showing a p-value < 0.05 and a FDR q-value < 0.20 were further considered. The difference of prevalence of Methanobacteriales before and after intervention was evaluated by chi-squared test between intervention groups and post-hoc stratification. DESeq2 library (version 1.14.1) [5] was used on OTUs raw count matrix derived from Qiime biom file. DESeq2 design parameter was set-up to consider the composition of the microbiota at baseline. OTU fold change significance between intervention groups (FMP, control) and post-hoc stratification was evaluated using Wald test. OTUs with a fold change supported by a p-value < 0.01 adjusted by false discovery rate were further considered. To quantify the proportion of microbiota regulated per group, read proportion for each significant OTU was computed per sample and summarized per intervention group and post-hoc stratification.

1. Le Nevé, B., et al., *Lactulose Challenge Determines Visceral Sensitivity and Severity of Symptoms in Patients With Irritable Bowel Syndrome*, in *Clinical Gastroenterology and Hepatology*. 2016 Feb;14(2):226-33.e1-3.

2. Le Nevé, B., et al., *A Combined Nutrient and Lactulose Challenge Test Allows Symptom-Based Clustering of Patients With Irritable Bowel Syndrome.* Am J Gastroenterol, 2013. **108**(5): p. 786-795.

3. Klindworth, A., et al., *Evaluation of general 16S ribosomal RNA gene PCR primers for classical and next-generation sequencing-based diversity studies.* Nucleic Acids Research, 2013. **41**(1): p. e1-e1.

4. Rognes, T., et al., *VSEARCH: a versatile open source tool for metagenomics.* PeerJ, 2016. **4**: p. e2584.

5. Love, M.I., W. Huber, and S. Anders, *Moderated estimation of fold change and dispersion for RNA-seq data with DESeq2.* Genome Biology, 2014. **15**(12): p. 550.
