# Supplementary material for: Fasting breath H2 and gut microbiota metabolic potential are associated with the response to a fermented milk product in irritable bowel syndrome
Source: PLoS One. 2019 Apr 4;14(4):e0214273. doi: 10.1371/journal.pone.0214273 (PMC6448848; doi:10.1371/journal.pone.0214273)

Research collaboration Danone – Sahlgrenska University Hospital, Gothenburg, Sweden: *Association between GI microbiota, low-grade inflammation and classical pathophysiological factors in patients with irritable bowel syndrome (IBS), and the role of treatment with* Activia®.

Magnus Simrén, Katarina Wilpart, Nora Khatai, Jenny Gunnarsson, Björn Lindkvist, Lena Öhman – Dept of Internal Medicine, Institute of Medicine, Sahlgrenska Academy, University of Gothenburg, Gothenburg, Sweden
Denis Guyonnet, Sophie Legrain-Raspaud, Danon, Paris, France
Joel Doré, INRA, Jouy-en-Josas, France

Background

There is a wide variety of symptoms in IBS, irritable bowel syndrome, and the symptom pattern varies within and between individuals. Patophysiological factors of importance have been identified, but none of these is present in all patients. Visceral hypersensitivity, abnormal motor and secretory function in the gastrointestinal (GI) tract, as well as psychological factors, are considered to be of relevance for IBS patients. However, the relative importance of specific factors for generation of symptoms is not well understood.

Abnormal autonomic and central nerve function have also been demonstrated in IBS, along with low grad inflammation within the GI tract, altered immune function, and changes in the gut flora. The importance of these factors in the development of the more established patophysiological factors, i.e. visceral hypersensitivity, and abnormal GI motor and secretory function, have not yet been studied. Moreover, the relevance of these recently discovered pathogenetic/pathophysiological factors for the symptom pattern in IBS remains largely unknown. There is also insufficient knowledge regarding the interaction between the autonomic and central nerve function and local pathogenetic factors within the gut, such as inflammation, altered immune function and aberrant composition/function of gut microbiota in IBS.

Based on the recent findings, regarding abnormalities in the gut flora in IBS, the use of probiotics as a treatment alternative has been tested, but the results are not consistent for all products that have been tested. The effects of some products are positive, but far from all the patients with IBS get adequate relief of their symptoms. The response to treatment with probiotics cannot be predicted with the methods available today; for example it is not possible to predict which patient who will respond to the treatment, and the mechanisms behind the symptom relief are unclear.

Questions

- Which factors are of importance for the symptom pattern in IBS?
    - Visceral hypersensitivity
    - Abnormal colonic motor function

    - Autonomic nerve dysfunction
    - Central nerve dysfunction (especially at the brain stem level)
    - Psychological factors
    - Coping
    - Demography (sex, age)
    - Immunological alterations in peripheral blood/intestine
    - Altered composition and function of the gut flora
    - Sexual/physical abuse
    - Pancreatic function

- How do patophysiological factors in IBS interact?
- Can we predict the treatment response to probiotics, based on the phenotype of the patient?
- Why do probiotics only lead to adequate relief in some of the patients with IBS?

Subjects:

- 150 patients with IBS according to Rome III criteria
- 50 healthy volunteers (not the treatment part of the study)

Inclusion criteria

- Signed written informed consent
- Age between 18 and 65 years old at baseline visit
- IBS according to the Rome III criteria
- Ability to understand and willingness to comply to the study procedures

Exclusion criteria

- Participation in another clinical study 1 month prior to screening visit and throughout the study
- Abnormal results on the screening laboratory tests, clinically relevant for study participation
- Other gastrointestinal disease(s) explaining the patient's symptoms, as judged by the investigator
- Other severe disease(s) such as malignancy, severe heart disease, kidney disease or neurological disease
- Symptoms indicating other severe disease(s) such as gastrointestinal bleeding, weight loss or fever
- Severe psychiatric disease
- Previous history of drug or alcohol abuse 6 months prior to screening
- Intolerance or allergy against milk products (only the treatment part of the study)
- Use of other probiotic products (according to sponsor's list) 2 weeks prior to the study and throughout the study
- Consumption of antibiotics 1 month prior to screening and throughout the study
- Consumption of cortisone, NSAID or other anti-inflammatory drugs on a regular basis 2 weeks prior to screening and throughout the study
- Pregnant or lactating or wish to become pregnant during the period of the study

Investigations

1. A structured investigation, covering the most important patophysiological factors in IBS and a thorough characterization of the symptom pattern of the patients.
2. A randomized, double blind placebo controlled study, evaluating the effects of probiotics (Bifidobacterium lactis, Activia®) on the symptom pattern in IBS, as well as measuring the effect of probiotics on the fermentation in the gut, immunological variables and the composition of the gut flora.

Visit 1

- The patients are thoroughly informed regarding the context of the study and written informed consent is obtained.

- Blood samples are drawn to rule out organic disease (hemoglobin, leukocytes, platelets, electrolytes, creatinine, liver tests, CRP, transglutaminase antibodies).

- Validated questioners are completed
    - IBS Symptom Severity Scale (IBS-SSS) – gastrointestinal and extra intestinal symptoms
    - Gastrointestinal Symptom Rating Scale – IBS (GSRS-IBS) – IBS specific GI-symptoms
    - Hospital Anxiety and Depression Scale (HAD) – general anxiety and depression
    - Visceral Sensitivity Index (VSI) – GI-specific anxiety
    - Patient Health Questionnaire-15 (PHQ-15) – severity of somatisation, depression, anxiety (panic anxiety, eating disorders and alcohol problems)
    - IBS Quality of Life Questionnaire (IBSQOL) – disease specific health related quality of life
    - Fatigue Impact Scale (FIS) – impact of fatigue on quality of life
    - Abuse Questionnaire – physical and sexual abuse
    - Sense of Coherence Index – sense of coherence, coping

- ABR (Auditory Brain stem Response audiometry) – brain stem audiometry measuring the electrical activity at level of the brain stem, while listening to different auditory stimuli (listening to sounds of clicks through headphones). Through this test the function of the brain stem can be measured, as well as its ability to mask the effect of disturbing noises etc.

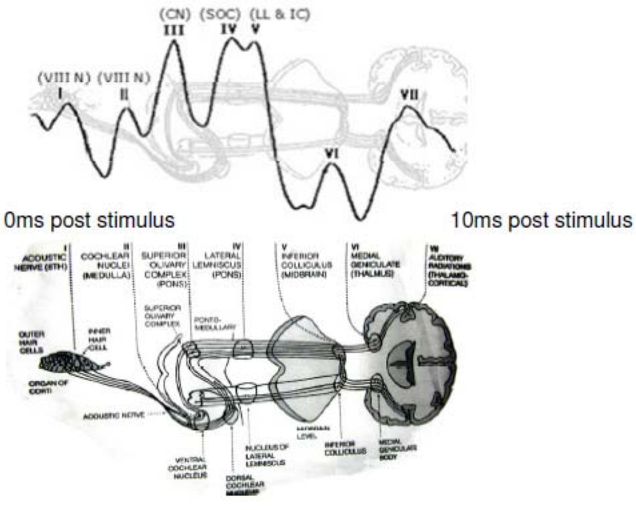

- 24h ECG recording – spectral analysis to evaluate the autonomic nerve function.

- Intestinal permeability test – the patient drinks a solution of water and sugars (50 g sucrose, 5 g mannitol, 10 g lactulose and 1 g sucralose in 200 ml tap water). Thereafter urine is collected during the following 9 h. The amount of sugars found in the urine is a measurement of the permeability in the intestine. The urine will be collected in separate portions, enabling measurement of the permeability in the small and large intestine separately.

- Registration of stool consistency and frequency over the following 2-3 weeks, until visit 3, using the Bristol Stool Form Scale (BSF)

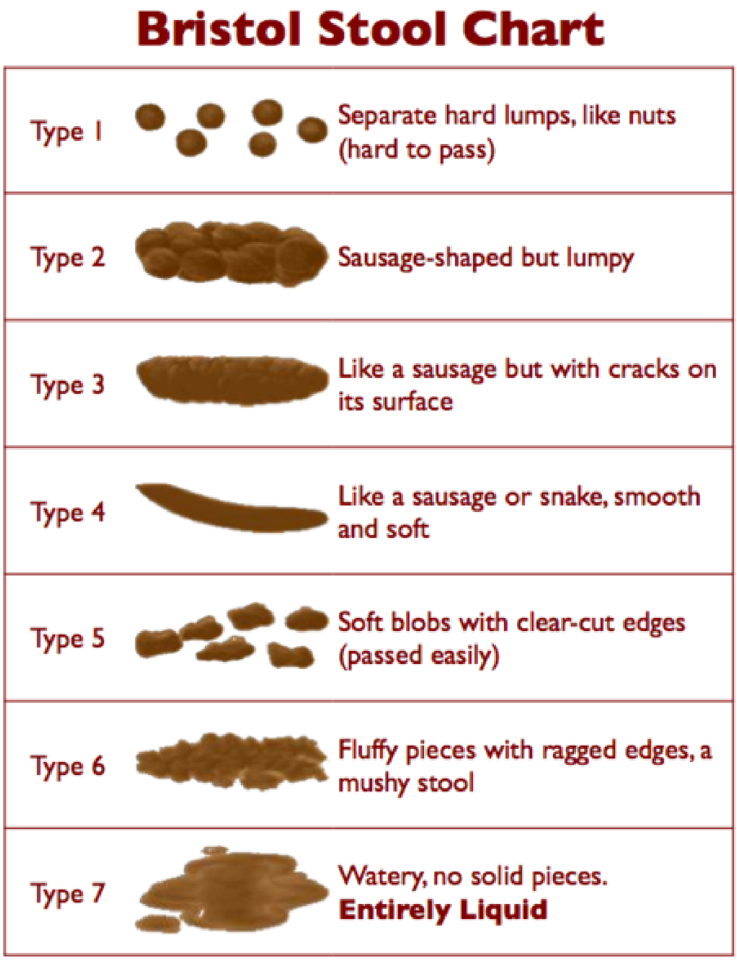

- Food diary – food intake over three consecutive days will be registered by the patient.

Visit 2

- Collection of urine samples
- The ECG recorder is returned by the patient.

Visit 3

- Salivary sample – test to measure the level of cortisol, an important stress marker.

- Oro-anal transit time – 60 radiopaque markers, possible to detect with x-ray, are ingested prior to the visit (10 markers/day during 6 days; fluoroscopy on day 7). This is a way to measure the oro-anal transit time, which mainly is determined by the colonic transit time. Thereby, this is an indirect measurement of the colonic motor function. Regional colonic transit can also be assessed by evaluating the localization of the markers remaining in the colon.

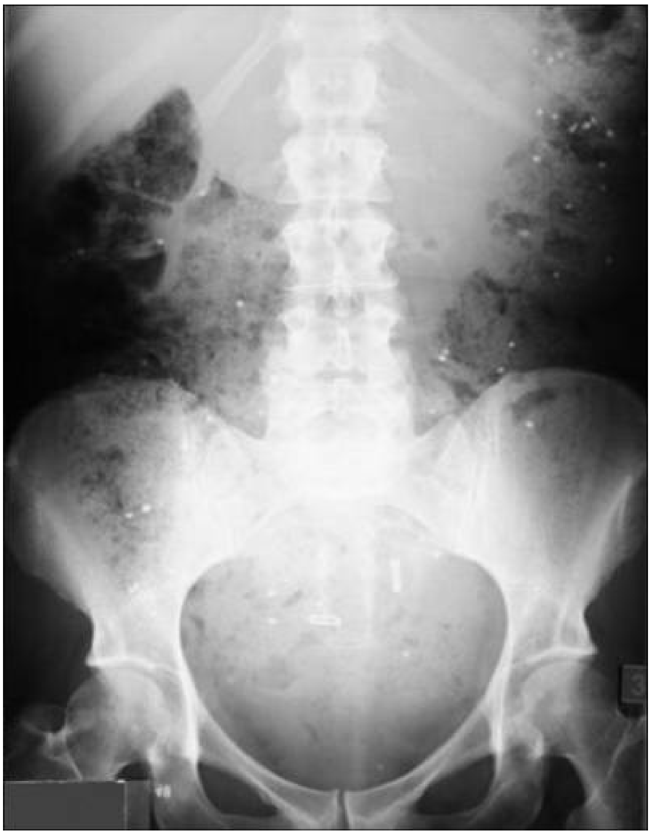

- Rectal sensitivity testing – balloon distension in the rectum by using a barostat, which makes it possible to inflate and deflate the balloon in a controlled manner and to measure pressure and volume in the balloon. During the distensions the sensitivity is determined by assessment of sensory thresholds, as well as by determination of the perceived intensity of different GI symptoms (VAS). Moreover, rectal compliance can be evaluated by evaluation of the pressure-volume relationship during the distensions.

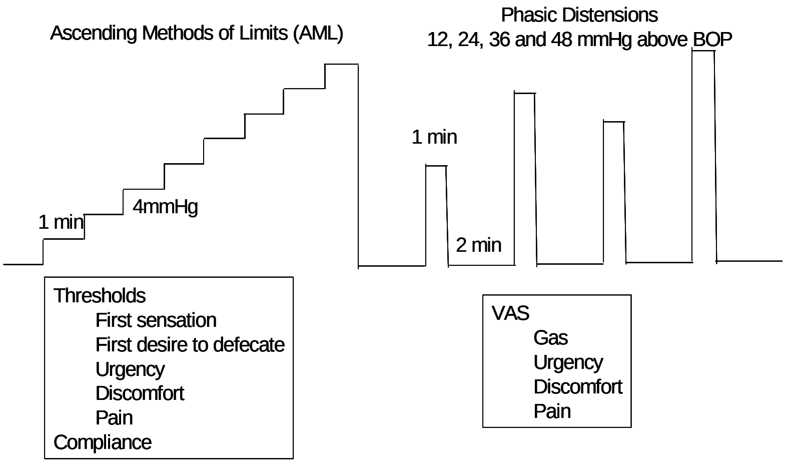

Visit 4

- Sigmoidoscopy with biopsies. Macroscopic evaluation of the mucosa of the large intestine, to rule out inflammatory bowel disease as a cause of the symptoms. Biopsies from the rectum and the sigmoid colon are taken for analysis of inflammatory cells, colonic immune function, and composition and function of the gut microbiota, using molecular techniques, such as 16sRNA.

- Stool sample – analysis of immunological and inflammatory markers in the faeces and of the composition and function of the luminal gut flora. A test for pancreatic function will also be taken (F-elastase).

- Blood samples – assessment of immune function, for example different pro- and anti-inflammatory interleukins and various food antibodies (IgE, IgG), is done to evaluate the "immunological status" of the patient and the propensity to react unfavorably when consuming certain food items.

- Salivary sample – test to measure the levels of cortisol, an important stress marker.

- Symptom protocol (IBS-SSS) and diary assessing stool consistency and frequency (Bristol Stool Form Scale (BSF)) are handed out. Symptoms and stools will be registered during 1-3 weeks and will be used as baseline assessment prior to the treatment period (see below).

Visit 5

- Lactulose challenge test – evaluation of symptoms and excretion of hydrogen and methane gas in the exhaled air after intake of a liquid meal (400ml Nutridrink®, 1.5 kcal/ml) containing 25 g lactulose. Hydrogen and methane gas in the exhaled air is a measurement of the degree of gut fermentation, and an indirect measure of bacterial overgrowth in the small intestine. This "challenge test" is a considered to be a more physiological way to study the sensitivity of the GI tract. The symptoms and excretion of gas will be registered during 4 h.

- Stool sample – analysis of immunological and inflammatory markers in the faeces and of the composition and function of the luminal gut flora.

- Salivary sample – test to measure the levels of cortisol, an important stress marker.

- Randomization. The patients will be randomized to receive a yoghurt containing probiotics (Bifidobacterium lactis, Activia®) or a yoghurt without probiotics (placebo), 125 g, twice daily, (morning and evening), treatment period 14 days. During the treatment period, the patients will record the severity of GI symptoms (Symptom protocol (IBS-SSS)), as well as stool consistency and frequency (Bristol Stool Form Scale (BSF)), using diary cards.

Visit 6

- Takes place on the last day of the treatment period.

- Collection of diaries – the patient is asked to complete diaries for another 14 days after the end of the treatment period.

- Lactulose challenge test is being repeated to evaluate potential differences in symptoms and excretion of hydrogen and methane gas in the exhaled air after intake of a liquid meal (400ml Nutridrink®, 1.5 kcal/ml) containing 25 g lactulose.

- Stool sample – analysis of immunological and inflammatory markers in the faeces and of the composition and function of the luminal gut flora.

- Blood samples – assessment of immune function, for example different pro- and anti-inflammatory interleukins and various food antibodies (IgE, IgG), is done to evaluate the "immunological status" of the patient and the propensity to react unfavorably when consuming certain food items.

Visit 7

- Summarizing visit – meeting a doctor specialized in functional GI-disorders. The results from the tests and analysis of the previous visits are being evaluated and any potential treatment is based on these results. The patients are offered a telephone based follow-up if they wish, otherwise they are referred back to primary care.

- Collection of diaries assessing GI symptoms after the treatment period.

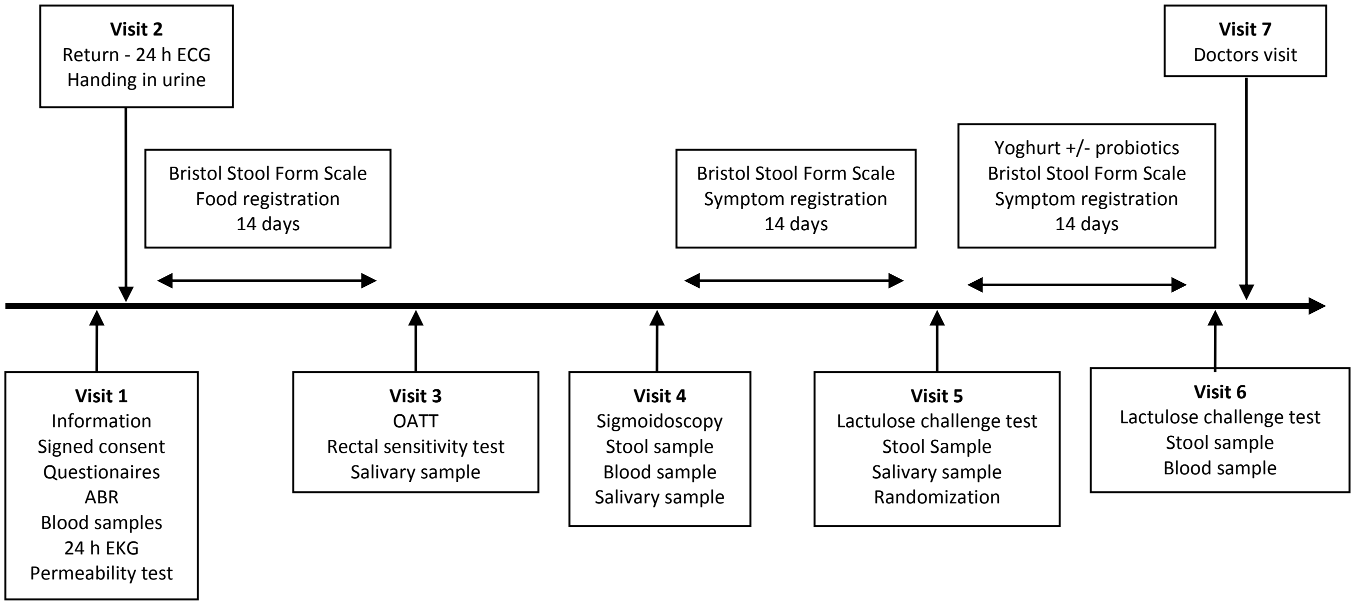

Supplement: S2 File — (PDF) [file pone.0214273.s004.pdf]
